# Utility of waist-to-height ratio in assessing the status of central obesity and related cardiometabolic risk profile among normal weight and overweight/obese children: The Bogalusa Heart Study

**DOI:** 10.1186/1471-2431-10-73

**Published:** 2010-10-11

**Authors:** Jasmeet S Mokha, Sathanur R Srinivasan, Pronabesh DasMahapatra, Camilo Fernandez, Wei Chen, Jihua Xu, Gerald S Berenson

**Affiliations:** 1 Center for Cardiovascular Health, Department of Epidemiology, Tulane University health Sciences Center, New Orleans, LA, USA

## Abstract

**Background:**

Body Mass Index (BMI) is widely used to assess the impact of obesity on cardiometabolic risk in children but it does not always relate to central obesity and varies with growth and maturation. Waist-to-Height Ratio (WHtR) is a relatively constant anthropometric index of abdominal obesity across different age, sex or racial groups. However, information is scant on the utility of WHtR in assessing the status of abdominal obesity and related cardiometabolic risk profile among normal weight and overweight/obese children, categorized according to the accepted BMI threshold values.

**Methods:**

Cross-sectional cardiometabolic risk factor variables on 3091 black and white children (56% white, 50% male), 4-18 years of age were used. Based on the age-, race- and sex-specific percentiles of BMI, the children were classified as normal weight (5th - 85th percentiles) and overweight/obese (≥ 85th percentile). The risk profiles of each group based on the WHtR (<0.5, no central obesity versus ≥ 0.5, central obesity) were compared.

**Results:**

9.2% of the children in the normal weight group were centrally obese (WHtR ≥0.5) and 19.8% among the overweight/obese were not (WHtR < 0.5). On multivariate analysis the normal weight centrally obese children were 1.66, 2.01, 1.47 and 2.05 times more likely to have significant adverse levels of LDL cholesterol, HDL cholesterol, triglycerides and insulin, respectively. In addition to having a higher prevalence of parental history of type 2 diabetes mellitus, the normal weight central obesity group showed a significantly higher prevalence of metabolic syndrome (p < 0.0001). In the overweight/obese group, those without central obesity were 0.53 and 0.27 times less likely to have significant adverse levels of HDL cholesterol and HOMA-IR, respectively (p < 0.05), as compared to those with central obesity. These overweight/obese children without central obesity also showed significantly lower prevalence of parental history of hypertension (p = 0.002), type 2 diabetes mellitus (p = 0.03) and metabolic syndrome (p < 0.0001).

**Conclusion:**

WHtR not only detects central obesity and related adverse cardiometabolic risk among normal weight children, but also identifies those without such conditions among the overweight/obese children, which has implications for pediatric primary care practice.

## Background

Childhood obesity is reaching epidemic proportions worldwide [1]. Although childhood obesity is a well recognized risk factor for developing cardiovascular disease and type 2 diabetes mellitus in adulthood, excess central (intra-abdominal) body fat distribution may be more related to these diseases than peripheral distribution [2]. Body Mass Index (BMI) is widely used as a measure to evaluate the impact of obesity on cardiovascular and metabolic risk factors, both in children and adults. However, in children, the BMI measures have to be expressed as *z *scores or percentiles relative to age and sex as BMI is strongly related to growth and maturation [3]. Moreover, BMI does not always relate to central obesity [4] and it cannot differentiate muscle mass from bone and fat mass [5]. Waist-to-Height Ratio (WHtR) has been proposed as an easily measurable anthropometric index for detection of central obesity and to assess associations between cardiometabolic risk factor variables and central intra-abdominal obesity [6-10]. Studies in adults have shown that it is possible to identify not only those with normal weight having an adverse cardiometabolic risk profile but also those with overweight/obese condition having normal metabolic risk profile [2,5,11]. Although studies relating BMI and WHtR to cardiovascular (CV) disease risk factors in children and adolescents are emerging [6,12-17], information is scant on the utility of WHtR in assessing the status of abdominal obesity and related cardiometabolic risk profile among normal weight and overweight/obese children. The objective of this current study was to examine this aspect in children enrolled in the Bogalusa Heart Study, a biracial (black-white) community-based study of the natural history of CV disease since childhood [18].

## Methods

### Study population

The present study sample was derived from 3238 children, 4-18 years of age (mean age 10.98 years, 50% male, 56% white). After excluding those with age-, race- and sex-specific BMI less than the 5^th ^percentile (n = 147), the subjects were divided into two groups: the normal weight group (n = 2581), with BMI between the 5^th ^and the 85^th ^percentiles; the overweight/obese group (n = 510), with BMI ≥ 85^th ^percentile. Using the previously recommended cut-point [6,8,14], children in each group were sub-divided based on their WHtR: centrally obese (WHtR ≥ 0.5) or non-centrally obese (WHtR < 0.5). The protocols of this study were approved by the Institutional Review Board of the Tulane University Health Sciences Center. Informed consent was obtained from all the participants, for those under 18 years of age, consent of a parent/guardian was obtained.

### General examination

Standardized protocols were used by trained examiners [19]. The subjects were instructed to fast overnight before screening. Anthropometric and blood pressure measurements were made in replicate, and the mean values were used. The BMI was calculated as the weight in kilograms divided by the square of height in meters. Waist circumference was measured midway between the lowest border of rib cage and the upper border of iliac crest while the child was standing. Blood pressure measurements were obtained using a mercury sphygmomanometer, on the right arm of the participants who were in a relaxed sitting position, by two trained observers, each recording three measurements. The first and the fourth Korotkoff phases were recorded for systolic and diastolic blood pressures respectively, and average levels were used for analysis.

### Laboratory Analyses

Serum cholesterol and triglyceride levels were assayed using enzymatic procedures (Abbott VP, North Chicago, Illinois) [20,21]. The levels of lipoprotein cholesterol were analyzed by a combination of heparin-calcium precipitation and agar-agarose gel electrophoresis procedures [22]. Plasma insulin measurements were obtained with the use of a commercial radio-immunoassay kit (Phadebas Insulin Kit; Pharmacia Diagnostics, Piscataway, NJ). Glucose was measured as part of a multiple chemistry profile (SMA 20) by a glucose oxidase method. The laboratory is monitored for precision and accuracy of lipid measurements by the Lipid Standardization and Surveillance Program of the Centers for Disease Control and Prevention (Atlanta, GA). Insulin resistance status was assessed as homeostasis model assessment of insulin resistance (HOMA-IR) according to the formula described [23]: insulin (μU/mL) × glucose (mmol/L)/22.5.

### Statistical Analysis

All statistical analyses were performed using Statistical Analysis Systems, version 9.1 (SAS Institute, Cary, North Carolina). Continuous variables were tested for normality using the Kolmogorov-Smirnov test. The values of insulin and HOMA-IR were log transformed to improve the normality of distribution. The differences in the mean values (mean ± standard error) of the cardiometabolic risk factor variables between the sub-groups were tested separately for each of the two groups (normal weight and overweight/obese) by analysis of covariance, adjusting for age, race and sex wherever necessary. In multivariate analyses, independent associations between the cardiometabolic risk factor variables and central obesity were examined for both groups using separate multivariate logistic regression analysis models, taking the presence or absence of central obesity (yes/no) as the dependent variable. Adverse levels were defined as age-, race- and sex- specific top tertiles of each cardiometabolic risk variable (except for HDL cholesterol) and the lower two tertiles were used as the reference group; bottom tertile versus the rest for HDL cholesterol. Categorical variables and prevalence rates were compared using χ^2 ^tests. With respect to metabolic syndrome, although there is a plethora of definitions used in studies of children, we used the one outlined by Cook et al [24].

## Results

**Figure 1:**
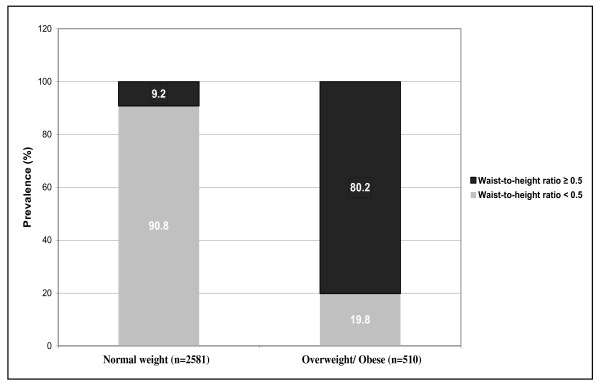
**Prevalence of Status of Central Obesity (Waist-to-Height Ratio <0.5 vs. ≥ 0.5) among Normal Weight and Overweight/Obese Children: The Bogalusa Heart Study**.

Figure 1 illustrates the prevalence of central obesity among normal weight and overweight/obese groups. Based on the WHtR (<0.5 vs. ≥ 0.5), 9.2% of children in the normal weight group had central obesity (n = 238), and 19.8% of the overweight/obese group did not (n = 101).

As shown in Table. 1, the centrally obese normal weight group, in addition to having significantly more males than females, showed higher age-, race- and sex- adjusted mean levels of systolic and diastolic blood pressures, mean arterial pressure (MAP), LDL cholesterol, triglycerides (TG), TG/HDL ratio, glucose, insulin and HOMA-IR, and lower mean levels of HDL cholesterol than the non-centrally obese normal weight group, whereas in the overweight/obese group, the levels of all the risk factor variables except diastolic blood pressure, MAP and glucose were significantly lower (higher levels of HDL cholesterol) in children without central obesity as compared to those who were centrally obese.

**Table 1:** Mean levels of cardiometabolic risk factor variables in normal weight and overweight/obese children by waist-to-height ratio: The Bogalusa Heart Study

	**NORMAL WEIGHT (BMI- 5th to 85th percentiles)**^**#**^			**OVERWEIGHT/OBESE (BMI **≥ **85th percentile)**^**#**^		
**Variable**	**Waist-to-Height Ratio**			**Waist-to-Height Ratio**		

	**<0.5 (n = 2343)**	≥**0.5 (n = 238)**	**p-value**	**<0.5 (n = 101)**	≥**0.5 (n = 409)**	**p-value**

**Age**	10.9 (0.07)	11.36 (0.23)	0.08	8.5 (0.34)	11.6 (0.16)	<0.0001

**Male %**	48.5	57.6	0.007	46.5	50.6	0.46

**White %**	53.4	74.0	<0.0001	33.7	60.1	<0.0001

**Systolic Blood Pressure (mm Hg)**	101.8 (0.16)	104.0 (0.51)	<0.0001	105.0 (0.88)	107.2 (0.41)	0.02

**Diastolic Blood Pressure (mm Hg)**	61.3 (0.14)	62.9 (0.45)	0.0008	64.4 (0.68)	64.9 (0.32)	0.47

**Mean Arterial Pressure (mm Hg)**	74.7 (0.13)	76.5 (0.42)	<0.0001	77.9 (0.63)	79.0 (0.30)	0.12

**LDL Cholesterol (mg/dl)**	100.2 (0.52)	110.4 (1.65)	<0.0001	103.8 (2.86)	111.7 (1.36)	0.01

**Triglycerides (mg/dl)**	72.1 (0.73)	95.4 (2.32)	<0.0001	88.4 (5.57)	108.5 (2.73)	0.002

**HDL Cholesterol (mg/dl)**	54.4 (0.23)	49.1 (0.75)	<0.0001	49.9 (1.03)	46.8 (0.49)	0.008

**TG/HDL Ratio**	1.4 (0.02)	2.2 (0.06)	<0.0001	1.9 (0.16)	2.5 (0.07)	0.003

**Glucose (mg/dl)**	79.4 (0.17)	81.9 (0.56)	<0.0001	81.1 (0.84)	81.9 (0.40)	0.39

Insulin (μU/ml)*	9.9 (0.13)	13.9 (0.43)	<0.0001	13.6 (1.37)	19.4 (0.63)	<0.0001

Insulin Resistance Index (HOMA-IR)*	1.9 (0.03)	2.8 (0.09)	<0.0001	2.8 (0.31)	3.9 (0.14)	<0.0001

Data presented as means (standard error) for continuous variables and percentages for categorical variables

*Values presented are the adjusted means, but log values were used for comparisons

^#^BMI percentiles were age-, race- and sex-specific

Continuous variables (except for age) were adjusted for age, race and gender.

HOMA-IR = Homeostasis model assessment of insulin resistance; TG/HDL Ratio = Triglyceride/HDL Cholesterol Ratio

The results of the multivariate logistic regression analysis are presented in Table 2. After adjusting for age, race and sex, the centrally obese normal weight group was 1.66, 1.47, and 2.05 times more likely than the non-centrally obese normal weight (reference group) to have significantly elevated levels of LDL cholesterol, triglycerides and insulin, respectively, and 2.01 times more likely to have significantly lower levels of HDL cholesterol. On the other hand, the odds of having adverse levels of HDL cholesterol and HOMA-IR were 0.53 and 0.27 times lower, respectively, in the non-centrally obese overweight/obese group versus the reference centrally obese overweight/obese group.

**Table 2:** Odds Ratios and 95% CI for adverse levels of cardiometabolic risk factor variables in normal weight and overweight/obese children: The Bogalusa Heart Study.

	**NORMAL WEIGHT (n = 2581) (BMI- 5th to 85th percentiles)**^**# **^**(Referenced to waist-to-height ratio <0.5)**			**OVERWEIGHT/OBESE (n = 510) (BMI **≥ **85th percentile)**^**# **^**(Referenced to waist-to-height ratio **≥**0.5)**		
Independent Variable (Top tertile vs. rest)*	**OR**	**95% CI**	**p-value**	**OR**	**95% CI**	**p-value**

**Mean Arterial Pressure (mm Hg)**	1.30	0.92-1.83	0.13	1.10	0.60-2.03	0.75

**LDL Cholesterol (mg/dl)**	1.66	1.18-2.32	0.003	0.61	0.34-1.10	0.10

**Triglycerides (mg/dl)**	1.47	1.02-2.11	0.03	0.59	0.32-1.07	0.08

**HDL Cholesterol (mg/dl)**	2.01	1.44-2.79	<0.0001	0.53	0.30-0.96	0.03

**Glucose (mg/dl)**	1.13	0.77-1.66	0.51	1.44	0.78-2.67	0.23

**Insulin (μU/ml)**	2.05	1.16-3.62	0.01	2.09	0.59-7.38	0.24

**Insulin Resistance Index (HOMA-IR)**	1.43	0.78-2.62	0.23	0.27	0.08-0.90	0.03

*Bottom tertile vs. the rest for HDL cholesterol. Tertiles were age-, race- and sex-specific. Models were age-, race- and sex-adjusted. All variables were included in both the models.

^# ^BMI percentiles were age-, race- and sex-specific

HOMA-IR = Homeostasis model assessment of insulin resistance; CI = Confidence Intervals

**Figure 2:**
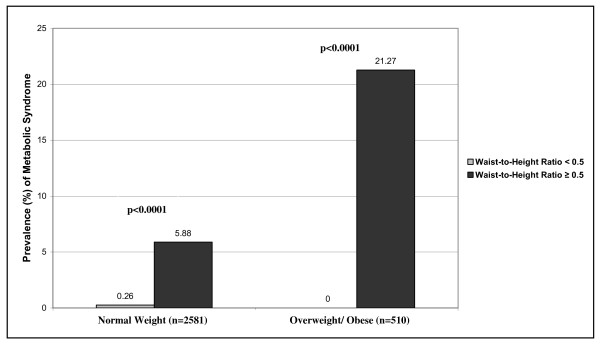
**Prevalence of Metabolic Syndrome in Normal Weight and Overweight/Obese Children According to Waist-to-Height Ratio: The Bogalusa Heart Study**.

Figure 2. demonstrates the prevalence of metabolic syndrome according to the WHtR groups in the normal weight and the overweight/obese children. As expected, 5.88% of the centrally obese normal weight subjects had metabolic syndrome as compared to only 0.26% of the normal weight without central obesity (p < 0.0001). Similarly, none of the overweight/obese children without central obesity showed the presence of metabolic syndrome whereas the prevalence in the centrally obese overweight/obese was as high as 21.27% (p < 0.0001).

As listed in Table 3., the prevalence of parental history of type 2 diabetes mellitus was significantly higher in individuals in the normal weight group having central obesity (11.71% vs. 6.63%, p = 0.007). The overweight/obese children without central obesity had a significantly lower prevalence with respect to parental history of hypertension (23.86% vs. 41.31%, p = 0.002) and type 2 diabetes mellitus (8.24% vs. 17.66%, p = 0.03) as compared to those with central obesity.

**Table 3:** Prevalence of parental history of hypertension, cardiovascular disease and type 2 diabetes in normal weight and overweight/obese children by waist-to-height ratio: The Bogalusa Heart Study.

	**NORMAL WEIGHT (n = 2581) (BMI- 5th to 85th percentiles)**^**#**^			**OVERWEIGHT/OBESE (n = 510) (BMI **≥ **85th percentile)**^**#**^		
**Prevalence (%)**	**Waist-to-Height Ratio**			**Waist-to-Height Ratio**		
	
	**<0.5**	≥**0.5**	**p-value**	**<0.5**	≥**0.5**	**p-value**

**Parental history of hypertension**	26.9	27.1	0.97	23.9	41.3	0.002

**Parental history of cardiovascular disease**	8.4	9.2	0.65	5.8	12.8	0.06

**Parental history of type 2 diabetes mellitus**	6.6	11.7	0.007	8.2	17.7	0.03

^# ^BMI percentiles were age-, race- and sex-specific

## Discussion

In this present cross-sectional, community-based study we used WHtR as a simple anthropometric index to identify the status of central (visceral) obesity and cardiometabolic risk factor profiles in groups of normal weight and overweight/obese children, selected based on traditional BMI criteria. Earlier studies in adults and children have supported the practicality of this convenient anthropometric index [6,8,10,15-17]. We found that while the normal weight children with central obesity had adverse levels of cardiometabolic risk factor variables as compared to those without central obesity, the overweight/obese without central obesity had significantly lower levels in relation to those with central obesity. These observations in children are in accordance with the emerging concepts that recognize subsets of obesity and support the existence of metabolically obese normal weight, and metabolically benign obesity phenotypes in adults [2,10,11,25]. These findings support the pathophysiologic role of intra-abdominal body fat distribution in determining interrelated cardiometabolic risk variables collectively recognized as metabolic syndrome [26-29]. It is now well recognized that central adiposity acts as a complex and highly active endocrine organ resulting in a variety of hormones and cytokines (tumor necrosis factor-α, interleukin-6 etc.), which in turn can play an important role in the dysregulation of inflammatory, metabolic and hemodynamic processes in the body through various mechanisms including hepatic lipogenesis and hepatic insulin resistance, release of free fatty acids from adipocytes, macrophage infiltration into the adipose tissue, adipose renin-angiotensin-aldosterone system and sympathetic nervous system activation and ectopic lipid storage [11,30,31].

Central obesity status regardless of normal weight or overweight/obesity condition in children is related to parental history of hypertension and type 2 diabetes, underscoring the familial nature of the relationship shown earlier in the Bogalusa Heart Study cohort [32,33]. Even though the overweight/obese without central obesity had a lower prevalence of parental history of CV disease the difference did not reach the level of significance. This may be explained by the fact that our study subjects are children and the age of onset of CV disease is higher than that of type 2 diabetes mellitus.

We believe that the above mentioned observations have important public health implications. The traditional cut-offs for BMI may underestimate the cardiometabolic risk in the normal weight and overestimate the same in the overweight/obese children while WHtR may be more sensitive in identifying the children at risk, especially at a population level, and provide a better estimate of the overall risk. As normal weight children may have already developed central obesity, health awareness efforts must include such children and advocate lifestyle changes, which may be easier to achieve in this group, before they develop overt obesity and its complications.

This community-based study had certain limitations in that it lacked direct assessments of pubertal status, body fat mass and distribution and in vivo insulin action. Instead, we used well-established surrogate measures that are simple and appropriate at the population level. Furthermore, this study being observational and cross-sectional in nature, could not address the issue of causality.

## Conclusion

The findings of the present study emphasize the utility of WHtR not only in detecting central intra-abdominal obesity and related cardiometabolic risk among normal weight children, but also in identifying those without central obesity and a healthy risk factor profiles among the overweight/obese children. Thus, WHtR has a potential for wider use as a simple measure to assess cardiometabolic risk in pediatric primary care practice.

## List of Abbreviations

BMI: Body Mass Index; WHtR: Waist-to-Height Ratio; LDL: Low Density Lipoprotein; HDL: High Density Lipoprotein; TG: Triglycerides; HOMA-IR: Homeostasis Model of Assessment of Insulin Resistance; MAP: Mean Arterial Pressure; CI: Confidence Intervals; OR: Odds Ratio

## Decleration of Competing interests

The authors declare that they have no competing interests.

## Authors' contributions

JSM made substantial contributions to the study design and acquisition of the data, performed statistical analysis, drafted the manuscript and made revisions. SRS participated in designing the study and revised the manuscript for intellectual content. PM assisted in statistical analysis and acquisition of the data. CF made contributions to the study design. WC made revisions to the manuscript and overlooked statistical analysis. JX carried out the laboratory analyses. GSB conceived of the study, revised the manuscript for intellectual content and gave final approval of the version to be submitted. All authors read and approved the final manuscript.

## Pre-publication history

The pre-publication history for this paper can be accessed here:

http://www.biomedcentral.com/1471-2431/10/73/prepub

## References

[B1] Must A Spadano J Coakley EH Field AE Colditz G Dietz WH The disease burden associated with overweight and obesity JAMA 1999 282 1523 9 10.1001/jama.282.16.1523 10546691

[B2] Stefan N Kantartzis K Machann J Schick F Thamer C Rittig K Balletshofer B Machicao F Fritsche A Häring HU Identification and characterization of metabolically benign obesity in humans Arch Intern Med 2008 168 1609 16 10.1001/archinte.168.15.1609 18695074

[B3] Kuczmarski RJ Ogden CL Guo SS Grummer-Strawn LM Flegal KM Mei Z Wei R Curtin LR Roche AF Johnson CL 2000 CDC Growth Charts for the United States: methods and development Vital Health Stat 112000 2002 1 190 12043359

[B4] Neovius M Rasmussen F Evaluation of BMI-based classification of adolescent overweight and obesity: choice of percentage body fat cutoffs exerts a large influence The COMPASS study. Eur J Clin Nutr 2008 62 1201 7 10.1038/sj.ejcn.1602846 17684527

[B5] Hall DM Cole TJ What use is the BMI? Arch Dis Child 2006 91 283 6 10.1136/adc.2005.077339 16551784 PMC2065990

[B6] Nambiar S Hughes I Davies PS Developing waist-to-height ratio cut-offs to define overweight and obesity in children and adolescents Public Health Nutr 2010 in press 20100388 10.1017/S1368980009993053

[B7] Higgins M Kannel W Garrison R Pinsky J Stokes J III Hazards of obesity-the Framingham experience Atca Med Scand 1988 723 Suppl 23 36 10.1111/j.0954-6820.1987.tb05925.x 3164971

[B8] Hsieh SD Muto T The superiority of waist-to-height ratio as an anthropometric index to evaluate clustering of coronary risk factors among non-obese men and women Prev Med 2005 40 216 20 10.1016/j.ypmed.2004.05.025 15533532

[B9] Bosy-Westphal A Geisler C Onur S Korth O Selberg O Schrezenmeir J Müller MJ Value of body fat mass vs anthropometric obesity indices in the assessment of metabolic risk factors Int J Obes 2006 30 475 83 10.1038/sj.ijo.0803144 16261188

[B10] Srinivasan SR Wang R Chen W Wei CY Xu J Berenson GS Utility of waist-to-height ratio in detecting central obesity and related adverse cardiovascular risk profile among normal weight younger adults (from the Bogalusa Heart Study) Am J Cardiol 2009 104 721 4 10.1016/j.amjcard.2009.04.037 19699351

[B11] Wildman RP Muntner P Reynolds K McGinn AP Rajpathak S Wylie-Rosett J Sowers MR The obese without cardiometabolic risk factor clustering and the normal weight with cardiometabolic risk factor clustering: prevalence and correlates of 2 phenotypes among the US population (NHANES 1999-2004) Arch Intern Med 2008 168 1617 24 10.1001/archinte.168.15.1617 18695075

[B12] Hara M Saitou E Iwata F Okada T Harada K Waist-to-height ratio is the best predictor of cardiovascular disease risk factors in Japanese schoolchildren J Atheroscler Thromb 2002 9 127 32 12226553 10.5551/jat.9.127

[B13] Maffeis C Banzato C Talamini G Obesity Study Group of the Italian Society of Pediatric Endocrinology and Diabetology Waist-to-height ratio, a useful index to identify high metabolic risk in overweight children J Pediatr 2008 152 207 13 10.1016/j.jpeds.2007.09.021 18206690

[B14] Goulding A Taylor RW Grant AM Parnell WR Wilson NC Williams SM Waist-to-height ratios in relation to BMI z-scores in three ethnic groups from a representative sample of New Zealand children aged 5-14 years Int J Obes (Lond) 2010 in press 20065976 10.1038/ijo.2009.278

[B15] Freedman DS Kahn HS Mei Z Grummer-Strawn LM Dietz WH Srinivasan SR Berenson GS Relation of body mass index and waist-to-height ratio to cardiovascular disease risk factors in children and adolescents: the Bogalusa Heart Study Am J Clin Nutr 2007 86 33 40 17616760 10.1093/ajcn/86.1.33

[B16] Savva SC Tornaritis M Savva ME Kourides Y Panagi A Silikiotou N Georgiou C Kafatos A Waist circumference and waist-to-height ratio are better predictors of cardiovascular disease risk factors in children than body mass index Int J Obes Relat Metab Disord 2000 24 1453 8 10.1038/sj.ijo.0801401 11126342

[B17] Kahn HS Imperatore G Cheng YJ A population-based comparison of BMI percentiles and waist-to-height ratio for identifying cardiovascular risk in youth J Pediatr 2005 146 482 8 10.1016/j.jpeds.2004.12.028 15812450

[B18] The Bogalusa Heart Study 20th anniversary symposium Am J Med Sci 1995 suppl 1 S1 S38 7503110 10.1097/00000441-199512000-00001

[B19] Berenson GS McMahan CA Voors AW Webber LS Srinivasan SR Frank GC Cardiovascular risk factors in children: the early natural history of athersclerosis and essential hypertension 1980 New York: Oxford University Press

[B20] Bucolo G David H Quantative determination of serum triglycerides by the use of enzymes Clin Chem 1973 19 476 82 4703655

[B21] Srinivasan SR Frerichs RR Webber LS Berenson GS Serum lipoprotein profile in children from a biracial community. The Bogalusa Heart Study Circulation 1976 54 309 18 181171 10.1161/01.cir.54.2.309

[B22] Srinivasan SR Berenson GS Lewis LA Opplt JJ CRC Handbook of electrophoresis (lipoproteins). In: Serum lipoproteins in children and methods for study 1983 3 Boca Raton (FL) CRC Press 185 203

[B23] Mathews DR Hosker JP Rudenski AS Maylor BA Trecher DF Turner RC Homeostatis model assessment: insulin resistance and β-cell function from fasting plasma glucose and insulin concentrations in man Diabetologia 1985 28 412 419 10.1007/BF00280883 3899825

[B24] Cook S Weitzman M Auinger P Nguyen M Dietz WH Prevalence of a metabolic syndrome phenotype in adolescents: findings from the third National Health and Nutrition Examination Survey, 1988-1994 Arch Pediatr Adolesc Med 2003 157 821 7 10.1001/archpedi.157.8.821 12912790

[B25] Dvorak RV DeNino WF Ades PA Poehlman ET Phenotypic characteristics associated with insulin resistance in metabolically obese but normal-weight young women Diabetes 1999 48 2210 4 10.2337/diabetes.48.11.2210 10535456

[B26] Nelson RA Bremer AA Insulin resistance and metabolic syndrome in the pediatric population Metab Syndr Relat Disord 2010 8 1 14 10.1089/met.2009.0068 19943799

[B27] Morrison JA Friedman LA Wang P Glueck CJ Metabolic syndrome in childhood predicts adult metabolic syndrome and type 2 diabetes mellitus 25 to 30 years later J Pediatr 2008 152 201 6 10.1016/j.jpeds.2007.09.010 18206689

[B28] Steinberger J Daniels SR Eckel RH Hayman L Lustig RH McCrindle B Mietus-Snyder ML Progress and challenges in metabolic syndrome in children and adolescents: a scientific statement from the American Heart Association Atherosclerosis, Hypertension, and Obesity in the Young Committee of the Council on Cardiovascular Disease in the Young; Council on Cardiovascular Nursing; and Council on Nutrition, Physical Activity, and Metabolism Circulation 2009 119 628 47 10.1161/CIRCULATIONAHA.108.191394 19139390

[B29] Bao W Srinivasan SR Berenson GS Persistent elevation of plasma insulin levels is associated with increased cardiovascular risk in children and young adults The Bogalusa Heart Study. Circulation 1996 93 54 9 10.1161/01.cir.93.1.54 8616941

[B30] Després JP Lemieux I Bergeron J Pibarot P Mathieu P Larose E Rodés-Cabau J Bertrand OF Poirier P Abdominal obesity and the metabolic syndrome: contribution to global cardiometabolic risk Arterioscler Thromb Vasc Biol 2008 28 1039 49 10.1161/ATVBAHA.107.159228 18356555

[B31] Kershaw EE Flier JS Adipose tissue as an endocrine organ J Clin Endocrinol Metab 2004 89 2548 56 10.1210/jc.2004-0395 15181022

[B32] Nguyen QM Srinivasan SR Xu JH Chen W Berenson GS Influence of childhood parental history of type 2 diabetes on the pre-diabetic and diabetic status in adulthood: the Bogalusa Heart Study Eur J Epidemiol 2009 24 537 9 10.1007/s10654-009-9372-5 19618280

[B33] McClain MR Srinivasan SR Chen W Steinmann WC Berenson GS Risk of type 2 diabetes mellitus in young adults from a biracial community: the Bogalusa Heart Study Prev Med 2000 31 1 7 10.1006/pmed.2000.0682 10896837

